# Efficacy and tolerability of subcutaneous repository corticotropin injection in refractory ocular inflammatory diseases

**DOI:** 10.1186/s12348-024-00428-8

**Published:** 2024-10-24

**Authors:** Negin Yavari, Hashem Ghoraba, Christopher Or, Zheng Xian Thng, S. Saeed Mohammadi, Irmak Karaca, Azadeh Mobasserian, Amir Akhavanrezayat, Anthony Le, Xun Lyu, Anadi Khatri, Woong Sun Yoo, Dalia El Feky, Ngoc Trong Tuong Than, Osama Elaraby, Aim-On Saengsirinavin, Xiaoyan Zhang, Frances Andrea Anover, Ankur Sudhir Gupta, Muhammad Sohail Halim, Louis A. Jison, Quan Dong Nguyen

**Affiliations:** 1 Department of Ophthalmology, Byers Eye Institute, Stanford University, 2452 Watson Court Suite 200, 94303 Palo Alto, CA USA; 2 National Healthcare Group Eye Institute, Tan Tock Seng Hospital, Nevona, Singapore; 3 University of Missouri Kansas City, Kansas City, MO USA

**Keywords:** Repository corticotropin injection, Adrenocorticotropic hormone, Uveitis, Scleritis

## Abstract

**Background:**

Repository corticotropin injection (RCI) has been suggested to exert immunomodulatory and anti-inflammatory effects in ocular inflammation. The index retrospective study aimed to evaluate the efficacy and tolerability of subcutaneous RCI in patients with active scleritis or uveitis.

**Main body:**

Medical records of patients who were diagnosed with different types of active scleritis or uveitis and received RCI for more than six months at a tertiary eye center were reviewed. Patient characteristics including age, sex, comorbidities, clinical findings, treatment details, and adverse events were recorded. A total of 17 eyes of 17 patients were included. Median age was 43 years old and 53% of patients were male. Mean treatment duration was 25.4 ± 15.5 months. Indications for RCI therapy were scleritis (7 anterior and 1 posterior) (47.8%), panuveitis (17.4%), retinal vasculitis (17.4%), chronic/recurrent anterior uveitis (13%), and posterior uveitis (4.35%). RCI was initiated at a dose of 40 to 80 units 3 times weekly. Given the adequate control of inflammation, RCI was successfully discontinued in four patients (23.5%). Prior to RCI therapy, 14 (82.3%) patients were on oral prednisone at an average of 10 mg daily (range 2.5–40 mg), and two (11.7%) patients discontinued prednisone immediately before initiating RCI due to side effects. After six months of therapy, the prednisone dose was reduced in four (23.5%) patients to an average of 3 mg daily (range 1–5 mg) and was stopped in eight (53%) patients. Concomitant immunomodulatory therapies (IMTs) included mycophenolate mofetil (23.5%) and methotrexate (23.5%), and adalimumab (23.5%). Ten patients were on IMTs prior to using RCI, and during the course of treatment, IMT was stopped in two patients and reduced in one. Side effects included insomnia (23%), hypertension (11.7%), lower extremity edema (11.7%), hyperglycemia (11.7%), weight gain (11.7%), and infection (5.8%).

**Conclusion:**

RCI may be considered as a potential therapy with acceptable tolerability for patients with non-infectious scleritis or uveitis.

## Introduction

Ocular inflammatory disease (OID) comprises a broad range of conditions, spanning from relatively common anterior uveitis to rare sight-threatening diagnoses like posterior uveitis and scleritis. Both infectious and non-infectious etiologies can result in OIDs with the latter being more common [1]. Different parts of the eye may be affected to varying degrees, and OID can manifest independently or as part of a systemic inflammatory condition which can pose potential life-threatening risks [2, 3].

The management of OIDs involve controlling inflammation, preventing vision loss, and reducing the occurrence of long-term complications while minimizing potential systemic or local adverse effects from therapy which can be quite challenging. Systemic steroids have been the primary treatment of OIDs, but their long-term use can be associated with numerous side effects including glucose intolerance, hypertension, edema, anxiety, osteoporosis, and infection [4–6]. Subcutaneous repository corticotropin injection (RCI) has emerged as a potential alternative treatment option for severe OID [7] and has been found to be effective in managing inflammatory ocular conditions with an acceptable safety profile [4]. RCI (Acthar^®^ Gel, Mallinckrodt, Dublin, Ireland), is a combination of naturally occurring analogues of adrenocorticotropic hormone and other pituitary peptides. It has received approval since 1952 from the US Food and Drug Administration (FDA) for several indications, including the treatment of specific patients with rheumatoid arthritis (RA), systemic lupus erythematosus (SLE), sarcoidosis, and noninfectious keratitis [8–11]. RCI not only stimulates an increase in production of natural steroid hormones, but also exhibits a distinctive non-steroidal anti-inflammatory effect by binding to melanocortin receptors (MCR). RCI binds to all MRCs and generates indirect anti-inflammatory effects through increasing release of endogenous steroid as well as direct immunomodulatory effects by inhibiting leukocytes, activity of proinflammatory mediators such as interleukin or tumor-necrosis factor, and inflammatory signaling [4, 12–14]. As a result, RCI can control OID without the usual side effects associated with high dose corticosteroids. Hence, we conducted a study to assess the efficacy and tolerability of RCI in managing patients with refractory uveitis or scleritis.

## Methods

### Study population

In this retrospective study, we examined patients with a diagnosis of active non-infectious uveitis or scleritis who underwent RCI treatment for a minimum of six months at a tertiary eye center from 2016 to 2023. The research was conducted in accordance with the Declaration of Helsinki, the United States Code of Federal Regulations Title 21, and the Harmonized Tripartite Guidelines for Good Clinical Practice (1996). As the study involved a retrospective review of patient records, an informed consent waiver was obtained for enrolled patients.

### Data collection and outcomes

Patient data were collected from our cohort, including demographic information such as age and sex, as well as clinical information such as coexisting comorbidities and type of OIDs that each patient had. Additionally, for those patients who had bilateral involvements, we only included the worse eye at the time of initiation of RCI therapy.

Regarding the RCI treatment itself, we recorded details about the dose and duration of treatment for each patient. Additionally, we documented any concurrent medications that the patients were taking, including steroids and other immunomodulatory agents.

Safety assessments were an important aspect of our study. We evaluated the tolerability of the drug and closely monitored any ocular or systemic adverse events that occurred during the course of treatment. Medical history and physical examination findings including vital signs and body weight were recorded on all patients at all visits. Additionally, blood tests such as complete blood counts and comprehensive metabolic panels were also reviewed. All systemic and ocular adverse events occurring during the treatment period were documented including hypertension, hyperglycemia, weight gain, insomnia, lower extremity edema, infection, glaucoma, and cataract.

If therapy discontinuation was necessary, we documented the reasons for discontinuation. RCI prescription was tailored according to each patient’s disease status; our routine approach was to start with 80 units of RCI three times/week, depending on factors such as concomitant medications. Subsequently, we tapered the RCI based on the patient’s response. There was no standardized protocol for the tapering process. Tapering was performed in a gradual manner based on the clinical response.

### Response versus non-response criteria

During each visit, patients were evaluated based on a set of predetermined criteria to evaluate their response to the RCI treatment (Table 1).

**Table 1:** Criteria of response and non-response in ocular inflammatory diseases

**Uveitis**
New active or worsening inflammatory lesions relative to baseline
Anterior chamber cell count is ≤ 0.5+
Vitreous Haze is ≤ 0.5+
Worsening of BCVA by ≥ 15 letters relative to best state achieved
New/worsening leakage in the fluorescence angiography
Increased macular thickness in the optical coherence tomography
**Scleritis** [15]
Grade 0: no scleral inflammation with complete blanching of vessels
Grade 0.5+: trace inflammation with minimally dilated deep episcleral vessels
Grade 1+: mild scleral inflammation with diffuse mild dilation of deep episcleral vessels
Grade 2+: moderate scleral inflammation with tortuous and engorged deep episcleral vessels
Grade 3+: severe scleral inflammation with diffuse significant redness of sclera ± obscuration of deep episcleral vessels with edema and erythema
Grade 4+: necrotizing scleritis with or without uveal show

### Statistical analysis

Descriptive statistics were calculated for the variables of interest, representing continuous variables as mean and standard deviation or median and range. Microsoft Excel (Version 16.77, Microsoft Corporation, Redmond, WA, USA) was used for statistical analysis.

## Results

### Demographic and baseline criteria

The study included a total of 17 eyes from 17 patients, with a median age of 43 years (ranging from 27 to 61 years), and 53% of the patients were male. The average duration of treatment was 25.4 ± 15.5 months, ranging from 10 to 68 months. The indications for RCI therapy were scleritis (seven eyes anterior scleritis, one eye posterior scleritis, 47.05%), panuveitis (three eyes, 17.64%), retinal vasculitis (three eyes, 17.64%), chronic/recurrent anterior uveitis retinitis (two eyes, 11.76%), and posterior uveitis (one eye, 5.88%) (Table 2). Detailed diagnoses of the OID is tabulated in Table 2. Among the study patients, there was variation in the dosing regimens of RCI, including different initial dosing regimens. Majority of patients received an initial dose of 80 units (*n* = 9) three times/week, followed by 40 units (*n* = 4) three times/week, 80 units (*n* = 3) two times/week, or 80 units (*n* = 1) five times/week. The duration of treatment varied as well. At the time of data collection, one patient was still undergoing treatment at a dose of 80 units three times/week, four patients were using 80 units two times/week, three patients were using a dose of 40 units three times/week, four patients were with using a dose of 40 units two times/week, and one patient was using 40 units once/week with an average treatment duration of 25.8 months. In four patients the dosage was gradually reduced from 190 units to 90.8 units in a mean duration of 51.7 weeks before tapering and completely discontinuing the therapy over a course of two weeks (Table 2).

There was no statistically significant change in the mean visual acuity of this cohort. Mean visual acuity (LogMAR) was 0.15 ± 0.25 pretreatment and 0.14 ± 0.24 posttreatment. Moreover, there were no significant changes in intraocular pressure (IOP), or changes in use of IOP lowering medication among the patients.

### Efficacy

Ocular inflammation remained stable in 10 eyes (58.8%) and showed improvement in seven eyes (41.2%). Overall, patients with scleritis showed more improvement with RCI therapy compared to patients with uveitis. Prior to RCI initiation, three patients exhibited peripheral vasculitis, and one of them experienced resolution of inflammation following RCI treatment. RCI therapy was successfully discontinued in four patients (three scleritis and one retinal vasculitis, 23.5%), after adequate control of inflammation. However, there was one patient with scleritis who experienced worsening of symptoms following cessation of RCI, necessitating use of other forms of immunomodulatory therapy (IMT) and inability to restart RCI due to a lack of insurance coverage. Among the other reasons leading to discontinuation of RCI therapy were insurance coverage in two patients and side effects such as uncontrolled hyperglycemia, hypertension, and lower extremity edema in two patients.

### Concomitant medications

Prior to initiating RCI therapy, 14 (82.3%) patients were receiving a daily oral prednisone dose of average 10 mg (range: 2.5–40 mg). After six months of RCI treatment, the dose was reduced to an average of 3 mg (range: 1–5 mg) in 4 (23.5%) patients, and it was completely discontinued in 9 (53%) patients. In two cases (11.7%), prednisone was stopped right before starting RCI due to intolerability of side effects from the corticosteroids. Concomitant IMTs included mycophenolate mofetil (23.5%) and methotrexate (23.5%), as well as biologics such as adalimumab (23.5%). Ten patients were already on IMTs prior to initiating RCI therapy. During the course of treatment, IMT was stopped in two patients, and reduced in one patient. One patient (5.8%) commenced adalimumab while using RCI (Table 2).

**Table 2:** Characteristics of patients with treatment for ocular inflammatory diseases and side effects

Case	Sex	Age	OID	Active in OD/OS	Duration, month	Initial oral steroids (mg)	IMT, biologics	Side Effects	Associated systemic condition	Comments
1	F	61	SLE associated anterior uveitis	OS	29	No	-	-	HTN, hypothyroidism, Raynaud’s syndrome	RCI started with a dose of 40U three times/week. Disease was stable after 7 months and the dose kept as is for maintenance.
2	M	18	HLA-B27 + associated anterior uveitis	OD	33	2.5	-	Insomnia, anxiety	Obesity	RCI started with a dose of 80U three times/week. Disease was stable after 29 months and the dose was kept as is for maintenance.
3	M	12	Idiopathic posterior uveitis	OD	16	4	MMF	-	-	RCI was started with a dose of 40U three times/week. The disease was stable after 8 months and the dose kept as is for maintenance.
4	M	40	Idiopathic panuveitis	OD	83	No	-	-	-	RCI started with a dose of 80U three times/week. The disease improved after 16 months and then dose was decreased to 80U twice/week and continued for maintenance.
5	F	35	Idiopathic panuveitis	OS	20	No	Adalimumab	-	Obesity, HTN	RCI started with a dose of 80U three times/week. The disease improved after 2 months and then the dose was decreased to 80U twice/week. However, due to insurance coverage, RCI was stopped and IMT initiated.
6	M	65	Idiopathic panuveitis	OS	32	15	MMF switched to adalimumab	Insomnia, weight gain	-	RCI started with a dose of 80U three times/week. The condition improved after 2 months and then the dose was tapered down to 80U twice/week. Due to adequate control of inflammation RCI was tapered and stopped.
7	M	64	Retinal vasculitis	OD	10	3	Adalimumab	Elevated BG, HTN, LEE, Infection	DMTII	RCI started with a dose of 80U three times/week. Even though the disease was stable, due to side effects the RCI was tapered and stopped.
8	F	27	IBD associated retinal vasculitis	OD	9	5	-	-	-	Prior to initiating the RCI, patient received IMT, however it was stopped. RCI started with a dose of 80U twice/week and increased to 80U three times/week based on the patient’s tolerance to control the inflammation. After four months, dose was reduced to 80U per week and continued for maintenance.
9	M	29	Idiopathic retinal vasculitis	OD	18	20	-	-	-	RCI was started at a dose of 80U three times/week, however due to insurance coverage RCI was stopped.
10	F	54	Idiopathic scleritis	OS	12	5	MTX	-	-	RCI was started at a dose of 80U three times/week and decreased to 80 U twice/week. RCI was stopped due to improvement and control of inflammation.
11	M	71	Scleroderma associated scleritis	OD	12	7.5	-	Insomnia, Elevated BG, LEE	-	RCI was started at a dose of 40U three time/week, however due to side effects it was stopped after a year. Scleritis was stable at the time of discontinuation. Patient was then lost to follow up.
12	F	70	Idiopathic scleritis	OS	35	9	MTX	-	-	RCI was started at a dose of 80U twice/week, then decreased after improvement to 40U weekly and continued for maintenance.
13	F	60	RA associated scleritis	OS	35	5	Adalimumab	Insomnia	Anemia	RCI was started at a dose of 80U three times/week, then tapered to 80U twice/week and continued for maintenance.
14	M	36	Celiac associated scleritis	OS	33	40	MMF	-	-	RCI was started at a dose of 80U twice/week, then tapered to 40U twice/week with improvement after one month and eventually due to control of inflammation RCI was tapered and stopped.
15	M	46	Idiopathic scleritis	OS	22	8	MTX switched to MMF	-	-	RCI was started at a dose of 80U three times/week, then after 10 months tapered to 40U three times/week. Due to control of inflammation RCI was tapered and stopped.
16	F	27	SLE associated scleritis	OS	39	5	MMF	Weight gain, HTN	Neutropenia	RCI was started at a dose of 80U five times/week, then tapered to 80U twice/week after 12 months and 40U twice/week after another year. Same dose was continued for maintenance.
17	F	60	RA associated scleritis	OS	24	2.5	MTX	-	-	RCI was started at a dose of 40U three/week, then decreased to 40U twice/week due to improvement after 6 months. Same dose was continued for maintenance.

mg = Milligram, IMT = Immunomodulatory therapy, SLE = Systemic lupus erythematosis, HTN = hypertension, RCI = Repository corticotropin injection, HLA = Human leukocyte antigen, M = Male, F = Female, OID = Ocular inflammatory diseases, OD = Right eye, OS = Left eye, MTX = Methotrexate, MM = Mycophenolate mofetil, LEE = lower extremity edema, DMTII = Diabetes mellitus type 2, RA = Rheumatoid arthritis

### Safety

**Fig. 1:**
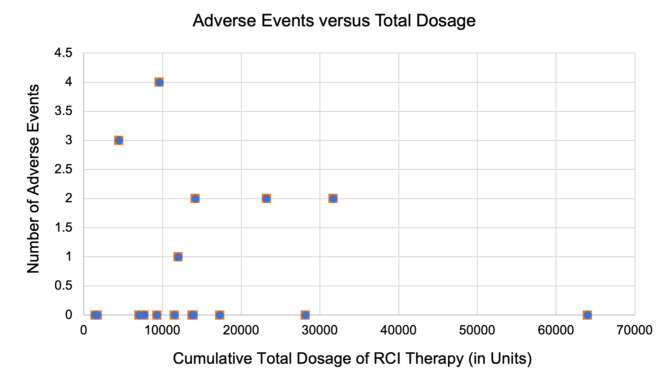
Number of adverse events during therapy with repository corticotropin injection (RCI) in cumulative total dosage (in Units)

No deaths and serious adverse events were reported in this case series. A total of 14 adverse events were reported, including insomnia (*n* = 4, 23%), hypertension (*n* = 2, 11.7%), lower extremity edema (*n* = 2, 11.7%), hyperglycemia (*n* = 2, 11.7%), weight gain (*n* = 2, 11.7%), anxiety (*n* = 1, 5.8%), and infection (*n* = 1, 5.8%) (Table 2; Fig. 1). Infection occurred after 10 months of initiating RCI in one patient. The patient had uncontrolled diabetes mellitus, which was not adequately controlled with increased hemoglobin A1C resulting in progression of nephropathy. During this period, the patient developed urinary tract infection which was managed with a course of antibiotics. RCI was subsequently discontinued due to the development of elevated blood glucose that was difficult to control. However, it should be noted that the OID was stable, and an alternative IMT was utilized. Notably, there were no significant ocular adverse events in the form of increased IOP, cataract progression, and infection in all patients throughout the course of treatment.

## Discussion

Despite being an FDA-approved medication for treating OIDs since 1952, the efficacy and tolerability of RCI has not been well established [16, 17]. Traditionally, the treatment options for OIDs have mainly involved the use of topical or systemic corticosteroids, along with steroid-sparing agents such as IMT and biologics [4, 16–18]. Based on the findings of our study, RCI may be an effective and tolerable treatment option for patients with various forms of uveitis and scleritis who require prolonged therapeutic interventions.

In our study, all patients initially received either corticosteroid treatment, conventional IMT, or biologic medications before starting RCI therapy. The decision to start RCI therapy was based on an inadequate response or unsatisfactory outcome of previous treatments or intolerance to previous therapy. Moreover, two patients had to stop taking prednisone due to significant, intolerable side effects prior to starting RCI. After 6 months of RCI therapy, nine patients were able to discontinue prednisone therapy and four had a dose reduction. Similarly, IMT was stopped in two patients and reduced in one patient. Throughout the study, four patients who discontinued their RCI dosage had no safety concerns, and we did not record any adverse events, whether systemic or local (dermal or ocular). Discontinuation was mainly due to lack of insurance coverage, However, nine patients still had mild activity and continued receiving RCI therapy. Out of these nine patients, four patients experienced a reduction in RCI treatment, three patients maintained the same dose, and one patient required an increased dosage from baseline. One scleritis patient who showed no improvement in disease activity, had to stop RCI and was successfully treated with rituximab infusions.

In a case report by Agarwal et al., they described a patient with panuveitis who initially received oral corticosteroids. Following an improvement in vitritis and retinal vascular leakage, to discontinue oral corticosteroids, the patient was enrolled in a trial (STOP-UVEITIS) to receive a course of treatment with tocilizumab. Significant improvement was achieved with tocilizumab. However, six months after completion of the study, inflammation recurred. Since the patient’s insurance did not cover additional tocilizumab therapy, RCI therapy was initiated which led to a notable reduction in retinal vascular leakage and inflammation after six weeks of treatment [7].

In a study conducted by Anesi et al., they evaluated the effect of RCI in 20 patients with non-infectious retinal vasculitis. After a mean duration of 5.8 years, at least 50% improvement was seen in 30 eyes, and nine eyes had complete resolution after a mean duration of 17.1 weeks [19]. Our results of patients with retinal vasculitis were also similar. All the patients showed improvement; however, two patients discontinued therapy.

In two studies, the clinical course of patients with non-infectious uveitis treated with RCI were evaluated. Nelson et al. did a medical review of 91 patients who were diagnosed with uveitis and all of them were receiving at least two types of medication including topical/oral corticosteroids, non-steroid eye drops, and biologics. After initiating RCI therapy, they demonstrated significant improvements in disease activity. They indicated that 76 patients (84%) improved, 15 patients (16%) were stable, and no patient had worsened [17]. Sharon et al. reported three cases of chronic, steroid-dependent uveitis who received RCI for more than a year. After a mean duration of 14 months, all three cases demonstrated improvement in their disease activity without any side effects [20]. These studies support the potential efficacy of RCI in the management of OIDs that are unresponsive or resistant to other treatments.

Prior to the initiation of RCI, it was noted that five eyes of three patients were already on topical IOP-lowering medication prior to the treatment, and their medication regimen was unchanged throughout the study. Importantly, no clinically significant increase in IOP was observed while the patients received RCI therapy. These findings align with a study conducted by Anesi et al., which also reported no clinically significant elevation in IOP measurements. While there were statistical changes in mean IOP from baseline to week 12 and week 24, these changes were minimal and suggested that significant or concerning elevation of IOP is rather uncommon when utilizing RCI therapy [19].

The adverse effects associated with RCI may be similar to those of systemic corticosteroids, encompassing fluid retention, imbalanced electrolytes, elevated blood glucose levels, systemic hypertension, mood alterations, weight gain, cushingoid features, and insomnia [21–23]. Anesi et al. observed a notable increase in the average weight at week 24 in comparison to week 12, implying that weight gain could be a potential side effect of RCI. The effect on weight is more likely to manifest after using the medication for more than three months, which holds implications for the potential long-term utilization of this treatment [19]. Oh et al. reported that four out of six patients in their cohort experienced severe side effects including hyperpigmentation, alopecia, and severe hypertension that led to the eventual discontinuation of therapy [24]. In our study, a total of 14 incidences of adverse events were observed. It is noteworthy that these side effects were predominantly concentrated in six patients, while eleven patients reported no side effects. Seven of these side effects occurred in two patients, potentially due to their underlying metabolic syndrome, which led to the development of uncontrolled blood glucose and blood pressure, and ultimately led to the discontinuation of therapy.

The most frequently encountered side effect in our series was insomnia. In their comprehensive Delphi Study, Nguyen et al. elaborated on the potential side effects of RCI therapy and their management, emphasizing the need for a thoughtful evaluation prior to starting RCI. Their study also suggests that specific patient related factors may play a significant role in the development of adverse events [4].

Apart from discontinuing therapy due to adverse effects, the cost of each vial of RCI compared to corticosteroids poses another challenge in its utilization. In fact, the study conducted by Oh et al. reported several instances where patients had to discontinue RCI therapy due to financial constraints [24]. In our study, two of our patients had to discontinue RCI therapy due to lack of insurance coverage for the medication. Overall, ophthalmologists should ensure the appropriate use of RCI in patients with OIDs, particularly due to potential complications and substantial cost burden on patients and/or healthcare systems [16].

Our investigation had certain limitations that should be acknowledged. Firstly, it was a retrospective study of patients with variable and non-homogenous etiology of OID, and analysis was done as a single cohort due to the small number of patients included. Since there is no established protocol for administering RCI, the dosing and duration of RCI usage are determined by clinical judgment, making it not possible to generalize one approach to every patient. Secondly, inclusion of patients on concomitant medications may have also influenced clinical outcomes, making it challenging to attribute them solely to RCI.

Finally, the small sample size in our study necessitates further investigations with larger cohorts to gain a more comprehensive understanding of the role of RCI in the management of different types of uveitis and OID.

## Conclusion

Treatment of minimally active or refractory OID is often challenging and often requires systemic steroids and IMTs, as steroid-sparing agents. The index study shows that RCI may be an efficacious and relatively safe, tolerable alternative for patients with recalcitrant non-infectious uveitis and scleritis or who are unable to tolerate other immunomodulatory agents.

Further research in a prospective, well-designed, randomized clinical trial is needed to establish a standardized guideline for the use of RCI in OID. Comparative studies using various dose-regimens can also be conducted to assess dose-response relationship and determine the most effective therapeutic dose. Moreover, management of concomitant medications such as corticosteroids and IMTs with RCI can be complex. Investigating the efficacy of combination therapy with RCI while taking concomitant anti-inflammatory medication should also be evaluated.

## Data Availability

No datasets were generated or analysed during the current study.

## Abbreviations

OID: Ocular inflammatory disease
RCI: Repository corticotropin injection
FDA: Food and Drug Administration
RA: Rheumatoid arthritis
SLE: Systemic lupus erythematosus
MCR: Melanocortin receptors
IOP: Intraocular pressure
IMT: Immunomodulatory therapy
